# Supplementary material for: Cryo-EM structure of the Saccharomyces cerevisiae Rpd3L histone deacetylase complex
Source: Nat Commun. 2023 May 27;14:3061. doi: 10.1038/s41467-023-38687-z (PMC10224958; doi:10.1038/s41467-023-38687-z)
Supplement: Supplementary file 1 — Supplementary Information [file 41467_2023_38687_MOESM1_ESM.pdf]

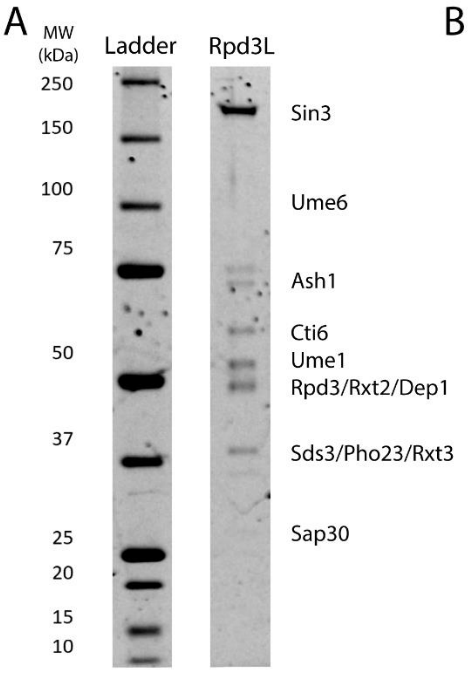

B

Accession Number	Protein	Molecular weight (kDa)	Spectrum count	Putative Stoichiometry
P22579	SIN3	175	2164	2
P32561	RPD3	49	804	2
Q03010	UME1	51	733	2
P31385	DEP1	47	492	1
Q08923	CTI6	57	397	1
Q07458	RXT3	34	322	1
P50947	PHO23	37	302	1
P38255	RXT2	49	279	1
P34233	ASH1	66	252	?
P40505	SDS3	38	189	1
P38429	SAP30	23	147	1
P10592	SSA2	69	119	-
P10591	SSA1	70	115	-
P35732	DEF1	84	54	-
P22202	SSA4	70	43	-
P11484	SSB1	67	43	-
P28003	FUN19	47	38	-
P10664	RPL4A	39	31	-
P38077	ATP3	34	23	-
P0CS90	SSC1	71	20	-
P29453	RPL8B	28	20	-
P39001	UME6	91	18	?
P27692	SPT5	116	17	-
P05739	RPL6B	20	16	-

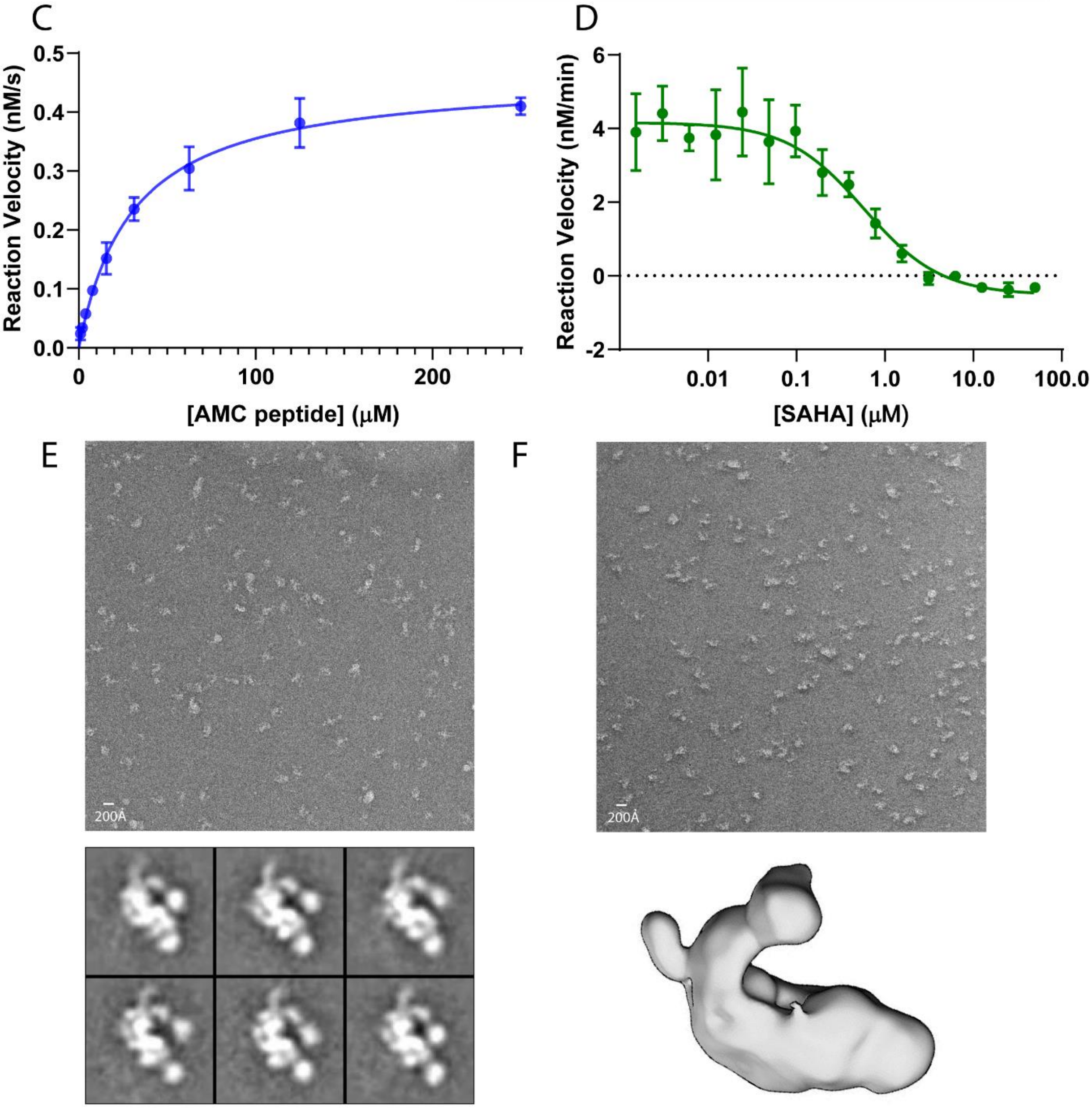

Supplementary Figure 1. Biochemical, mass spectrometric, and EM analysis of the Rpd3L complex.

A. SDS-PAGE analysis of the Rpd3L complex following tandem affinity purification (TAP) of the Rxt2 subunit. Protein bands were visualized by silver staining.

B. Mass spectrometric analysis of the purified sample in panel A revealing the constituents, spectral counts, and stoichiometry. Mass contribution based on putative stoichiometry, in parentheses are modeled masses.

C. Deacetylase assays demonstrating Michaelis-Menten kinetics for the purified Rpd3L complex. Data shown are the mean (center) and standard deviation (error bars) of three independent measurements. Source data are provided as a Source Data file.

D. Inhibition of deacetylase activity in the Rpd3L complex by suberoylanilide hydroxamic acid (SAHA). Data shown are the mean (center) and standard deviation (error bars) of three independent measurements. Source data are provided as a Source Data file.

E. Electron micrograph (*top*) and 2D class averages (*bottom*) following negative staining of the sample in panel A.

F. Electron micrograph at 40° tilt (*top*) and 3D reconstruction (*bottom*) following negative staining of the sample in panel A.

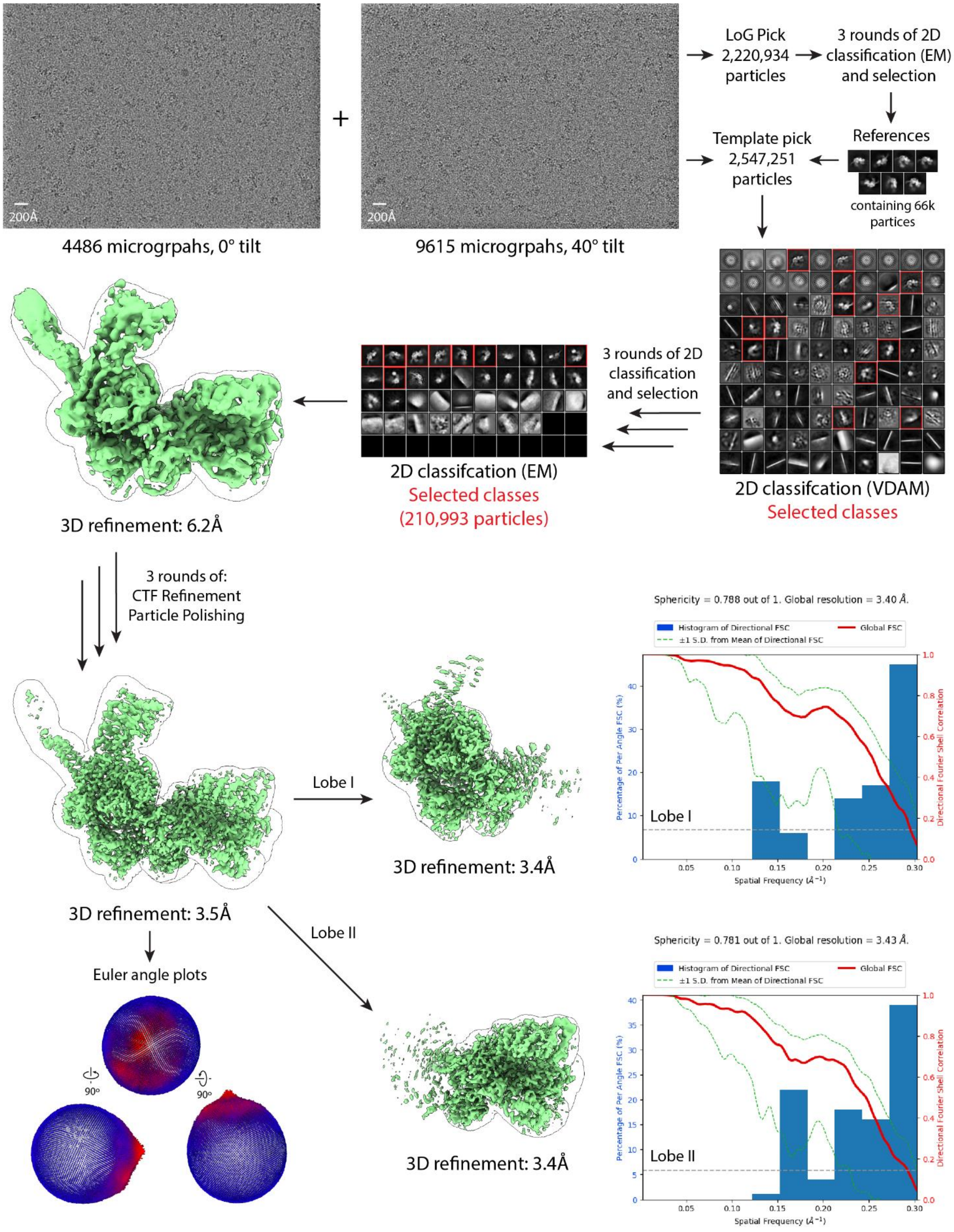

Supplementary Figure 2. Cryo-EM data processing and analysis for the Rpd3L complex.

Cryo-EM data collection and processing for Rpd3L. Particles from class averages outlined in red were selected for further processing. The final global refinement yielded a map with an overall resolution of 3.5 Å. To improve map quality of Lobes I and II, both were separately refined to a resolution of 3.4 Å. Euler angle distribution plots are shown in three orthogonal views (below final global 3D refinement). 3D FSC plots for focus refined regions of Lobes I and II (*bottom right*).[1]

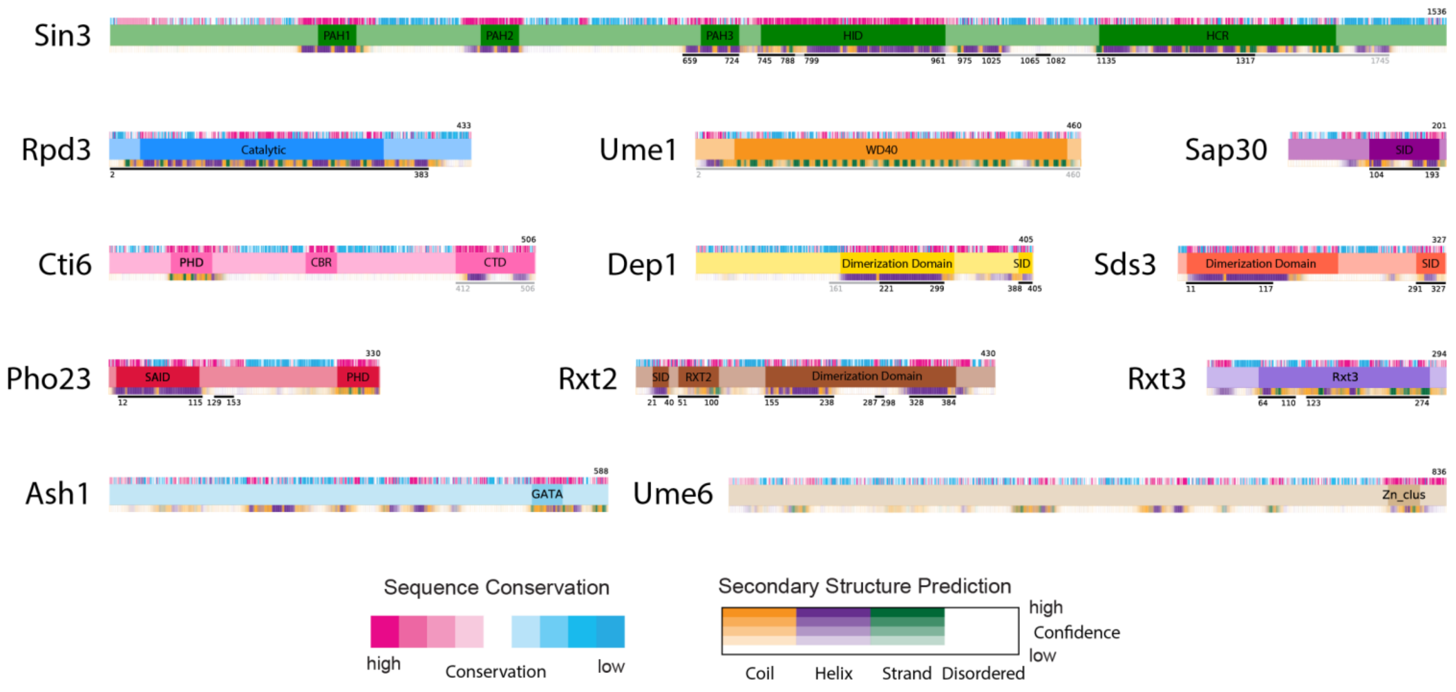

Supplementary Figure 3. Domain maps of subunits of the Rpd3L complex.

In each map, from top to bottom, residue- or domain-level data from ConSurf[2,3] (sequence conservation), PFAM[4] (protein domain family), and PSIPRED/DISOPRED3[5-7] (secondary structure/disorder) are presented. The coloring scheme for sequence conservation and secondary structure/disorder is indicated in the legend at the bottom of the figure.

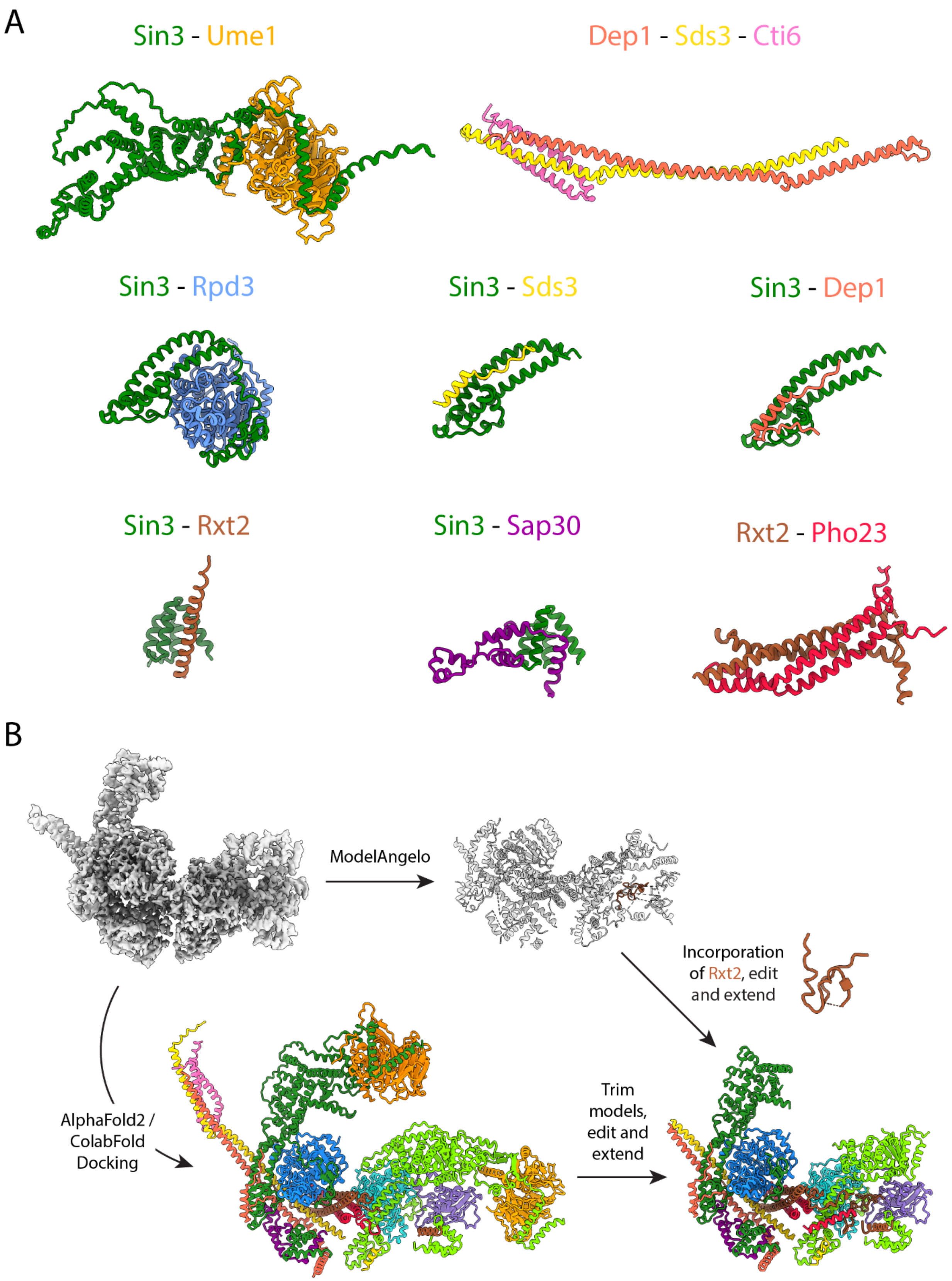

Supplementary Figure 4. Model building workflow for the Rpd3L complex.

A. Atomic models of various binary and ternary sub-complexes predicted using AlphaFold2-multimer.

B. The predicted models from panel A were docked into the cryo-EM map and the map itself was iteratively refined and enhanced to allow *de novo* chain tracing and model building for subunits (*e.g.*, Rxt2) with novel folds or engaged subunits through irregular backbone conformations.

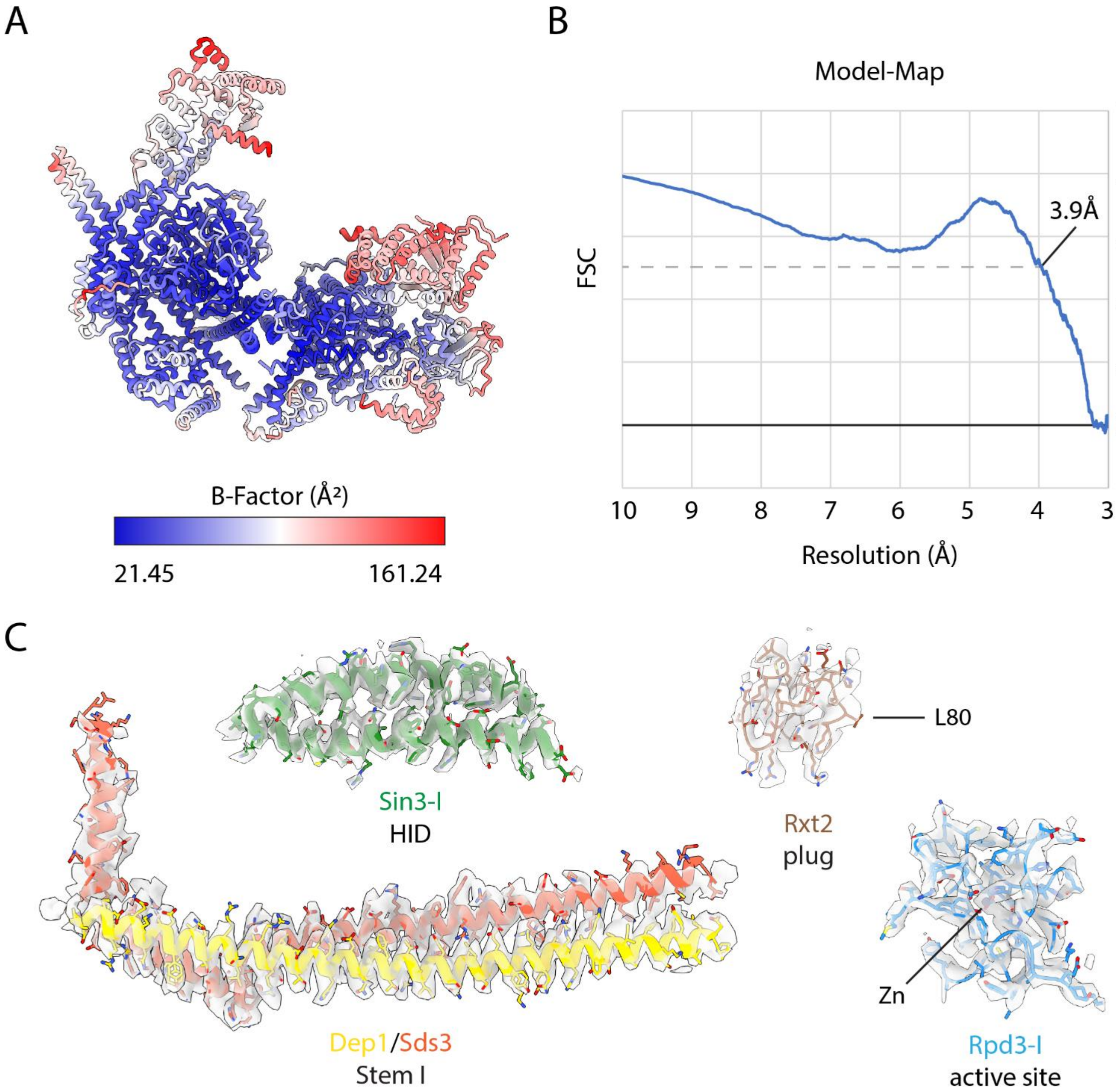

Supplementary Figure 5. Model validation for the Rpd3L complex.

A. Structure of the Rpd3L complex colored according to refined *B*-factors values.

B. A model-map FSC curve.

C. Transparent regions of the consensus map of the Rpd3L complex, segmented, with atomic models shown.

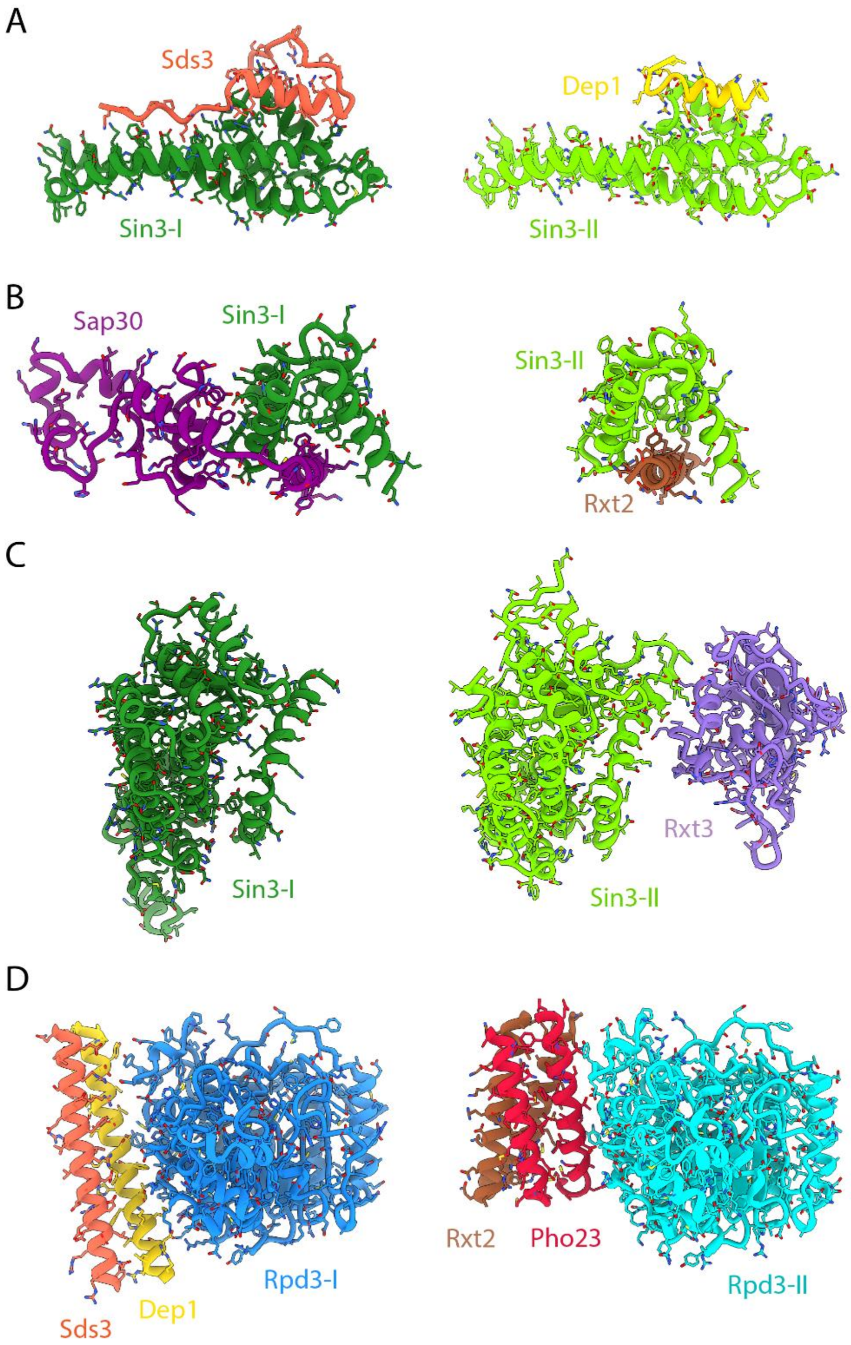

Supplementary Figure 6. Differential interactions involving Sin3 and Rpd3 in the two lobes.

A. Sds3 engages the Sin3-I HDAC interaction domain (HID) through a bipartite motif comprising an extended segment followed by a helix as described previously for the mammalian sub-complex, Dep1 interacts only through a helical segment because a portion of the HID interface is partially occluded by a Pho23 helix (not shown).

B. Both Sap30 and Rxt2 engage with the partially exposed hydrophobic cleft in the PAH3 domain of Sin3. Unlike Rxt2, Sap30 additionally engages another Sin3 surface comprising two helices as also described previously for the mammalian sub-complex.

C. Rxt3 engages the highly conserved region (HCR) of Sin3-II at the C-terminus. The equivalent region in Sin3-I is unoccupied.

D. Dep1 and Pho23 engage a comparable surface of the catalytic domain of Rpd3 in the two lobes. Both sets of interactions rely on a single helix in the coiled-coil and four-helix bundle formed with Sds3 and Rxt2, respectively.

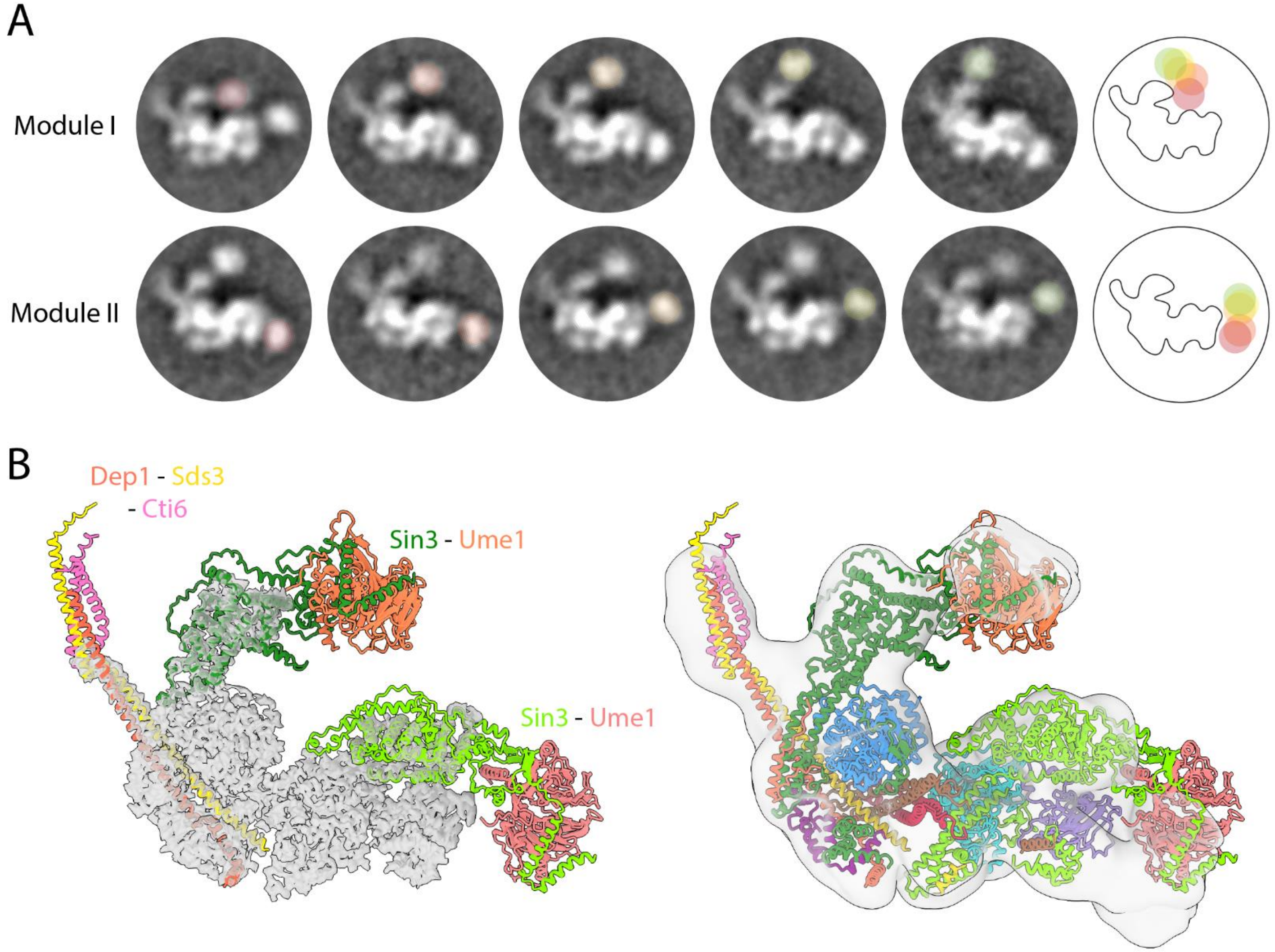

Supplementary Figure 7. Flexible modules within the Rpd3L complex.

A. Selected 2D class averages from electron micrographs following negative staining of the sample illustrating differences in the locations of Modules I (*top*) and II (*bottom*) relative to the structured core. The modules are colored differently in these micrographs and the positional disorder is summarized in the cartoons on the right.

B. *Left*: AlphaFold2-multimer models for the ternary sub-complex formed by Dep1, Sds3, and Cti6 at the tip of Stem I and for the binary sub-complex formed by Sin3 and Ume1 at the tips of Lobes I and II superimposed on top of the cryo-EM map contoured at a high threshold. *Right*: A hybrid model of the cryo-EM and AlphaFold2-multimer structure of the Rpd3L complex superimposed on the cryo-EM map contoured at a much lower threshold.

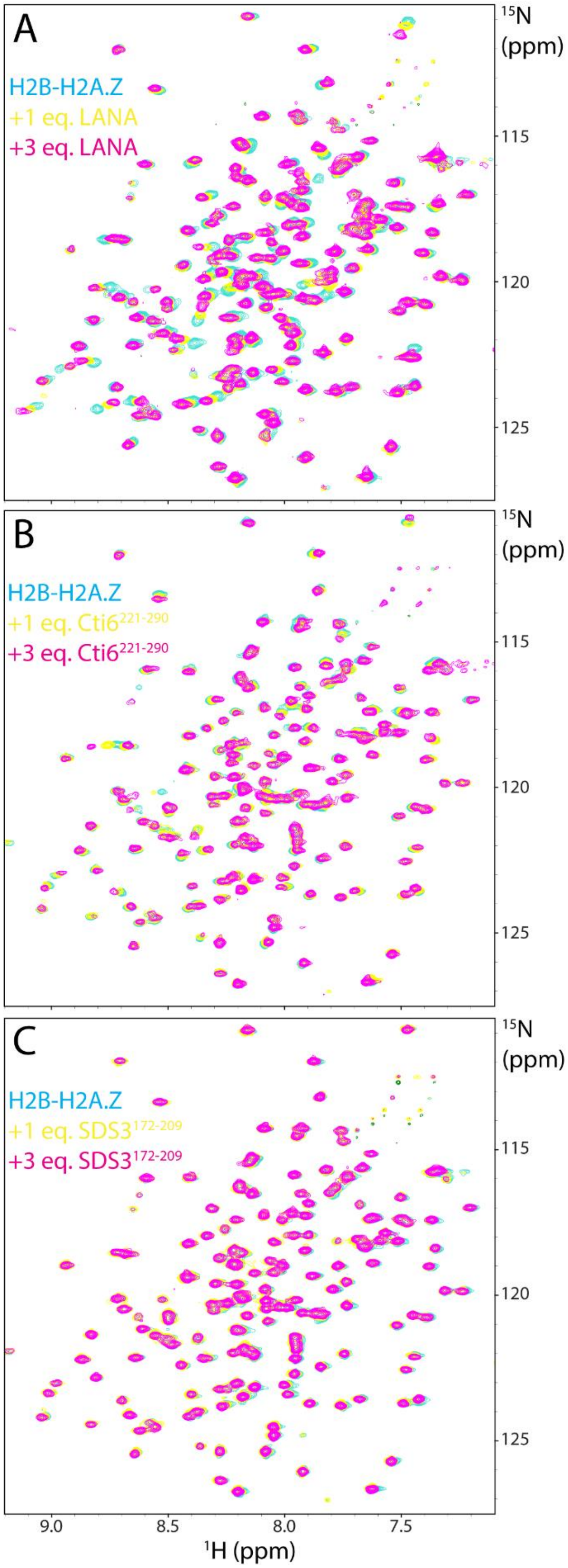

Supplementary Figure 8. A construct bearing the CBR region in Cti6 interacts with the H2A-H2B dimer. ^{15}N-labeled H2B-H2A.Z fusion protein was titrated with the indicated amounts of (**A**) the LANA peptide, (**B**) the Cti6$^{221\text{-}290}$ construct, and (**C**) the SDS3$^{172\text{-}209}$ peptide. Spectra were processed using identical parameters and contoured for the overlays after taking dilution caused by the titrants into account to facilitate cross-spectral comparisons.

A

Yeast	Human
Sin3	SIN3A/B
Ume1	RBBP4/7
Rpd3	HDAC1/2
Sds3	SDS3
Sap30	SAP30
Pho23	ING1B/2
Dep1	BRMS1
Rxt2	SAP130
Cti6	ARID4A
Rxt3	-

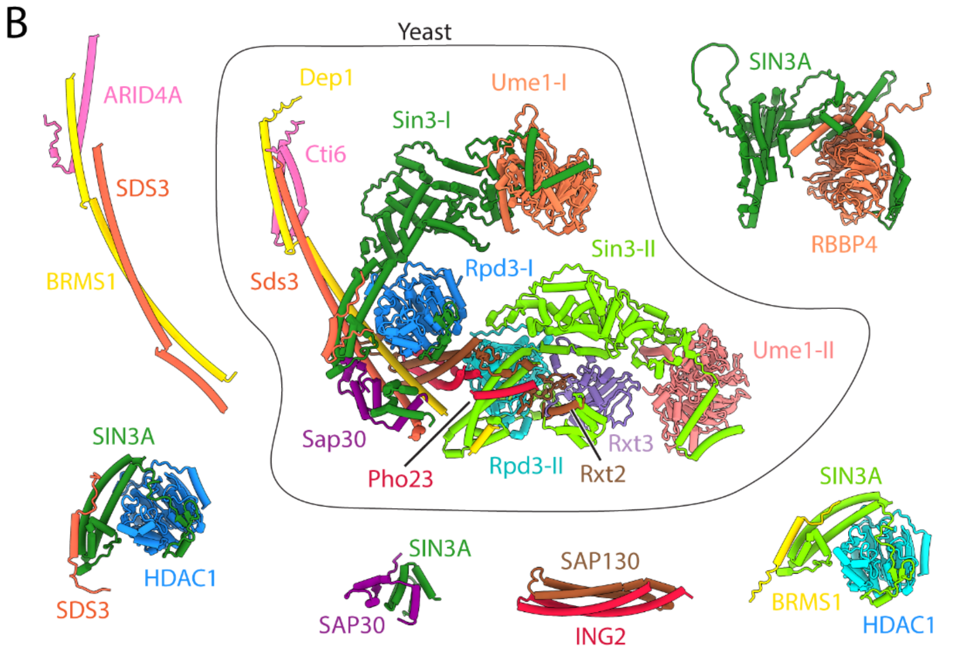

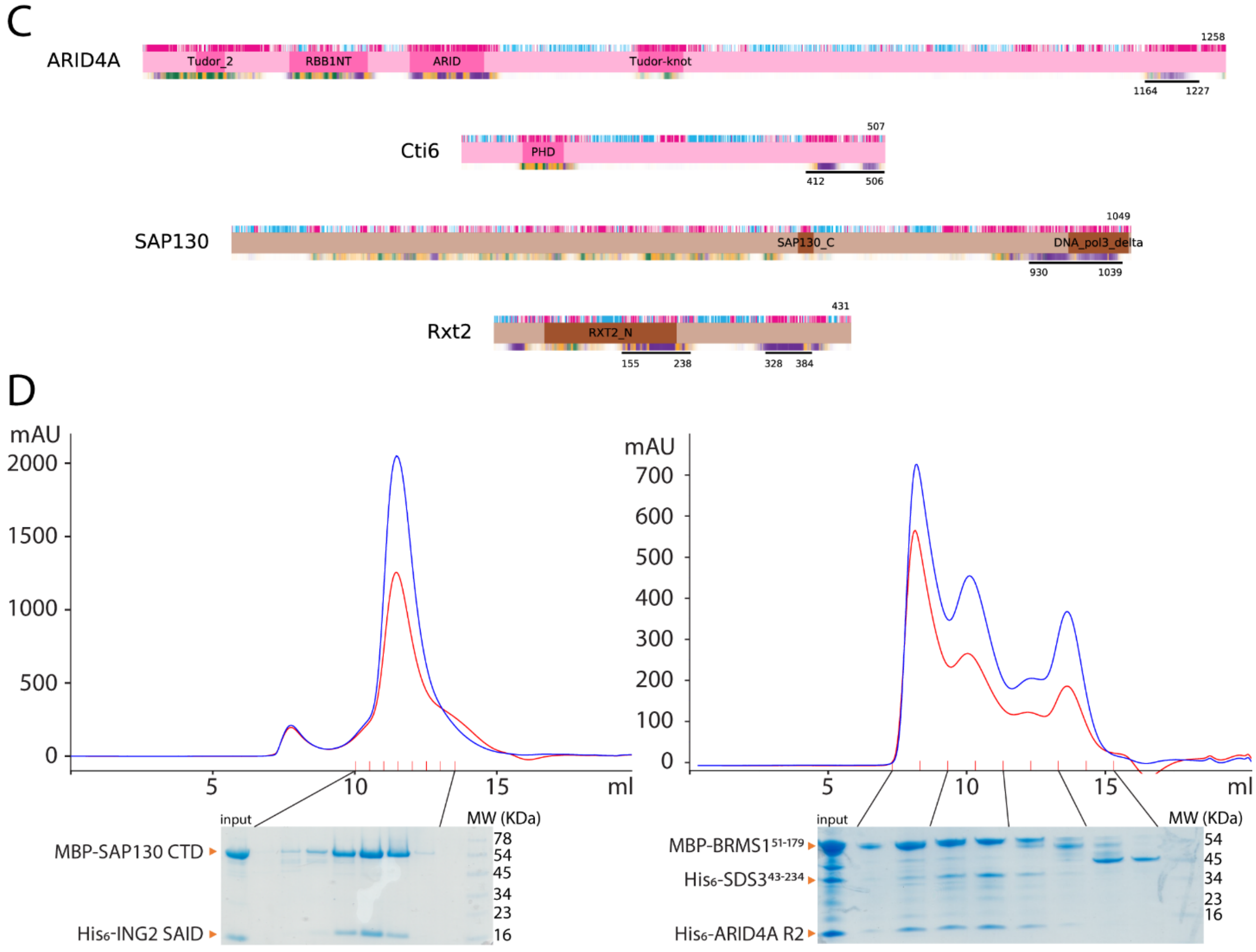

Supplementary Figure 9. The yeast Rpd3L and mammalian Sin3L/Rpd3L complexes share homologous/analogous subunits.

A. A table listing equivalent subunits in the yeast and mammalian complexes.

B. At center is the yeast Rpd3L complex and surrounding are the AlphaFold2-multimer models for human subunits that color the same as their yeast equivalents.

C. Domain graphs of ARID4A and Cti6 and SAP130 and Rxt2. Regions marked with black bars are those that appear to be functionally equivalent.

D. Results of size-exclusion chromatography (SEC) assays of sub-complexes formed by SAP130 and ING2 and ARID4A, BRMS1, and Sds3. The indicated proteins were co-expressed or combined after expression as His_6- or maltose-binding protein (MBP)-tagged proteins in bacteria, purified via Ni^{2+}-affinity chromatography, and subjected to SEC assays. The chromatograms monitoring absorbance at 280 nm (blue) and 260 nm (red) are shown. SDS-PAGE analyses of fractions from these assays followed by colloidal Coomassie Brilliant Blue staining. SAP130 and ING2 co-elute together, establishing a direct interaction between the interacting domains. ARID4A co-elutes with MBP-tagged BRMS1 and also with the heterodimeric complex formed by MBP-BRMS1 and Sds3, establishing ternary complex formation involving these subunits. SEC assays were performed at least twice and similar results were obtained each time.

Supplementary References

1. Tan YZ, *et al*. Addressing preferred specimen orientation in single-particle cryo-EM through tilting. *Nat Methods* **14**, 793-796 (2017).

2. Ashkenazy H, *et al*. ConSurf 2016: an improved methodology to estimate and visualize evolutionary conservation in macromolecules. *Nucleic Acids Res* **44**, W344-350 (2016).

3. Landau M, *et al*. ConSurf 2005: the projection of evolutionary conservation scores of residues on protein structures. *Nucleic Acids Res* **33**, W299-302 (2005).

4. El-Gebali S, *et al*. The Pfam protein families database in 2019. *Nucleic Acids Res* **47**, D427-D432 (2019).

5. Buchan DW, Minneci F, Nugent TC, Bryson K, Jones DT. Scalable web services for the PSIPRED Protein Analysis Workbench. *Nucleic Acids Res* **41**, W349-357 (2013).

6. Buchan DWA, Jones DT. The PSIPRED Protein Analysis Workbench: 20 years on. *Nucleic Acids Res* **47**, W402-W407 (2019).

7. Jones DT, Cozzetto D. DISOPRED3: precise disordered region predictions with annotated protein-binding activity. *Bioinformatics* **31**, 857-863 (2015).
