# Supplementary material for: Cryo-EM structure of the Saccharomyces cerevisiae Rpd3L histone deacetylase complex
Source: Nat Commun. 2023 May 27;14:3061. doi: 10.1038/s41467-023-38687-z (PMC10224958; doi:10.1038/s41467-023-38687-z)
Supplement: Supplementary file 3 — Description of Additional Supplementary Files [file 41467_2023_38687_MOESM3_ESM.pdf]

File name: Supplementary Data 1

Description: An extended Rpd3L model as seen in Fig. 4B.
